# 

**Published:** 1815-04

**Authors:** 


### MONTHLY CATALOGUE OF MEDICAL BOOKS.

A TREATISE on Fever, with Observations on the Practice adopted for its Cure in the Fever Hospital and House of Recovery, in Dublin. By William Stoker, M.D. 8vo.—Longman and Co.

Researches about Atmospheric Phenomena. By Thomas Forster, F.L.S. Second edition corrected and enlarged; with a Series of Engravings, illustrative of the Modifications of the Clouds, &c. 8vo.—Baldwin and Co.

Practical Observations on Necrosis of the Tibia; illustrated with Cases and a Copper Plate. By Thomas Whately, Esq.

A Treatise on the Diseases of Arteries and Veins, containing the Pathology and Treatment of Aneurisms and Wounded Arteries. By Joseph Hodgson, Esq. 8vo.—Underwood.

Engravings intended to illustrate some of the Diseases of Arteries. By Joseph Hodgson, Esq. 4to.—Underwood.

Mineralogical Nomenclature, alphabetically arranged, with Synoptic Tables of the Chemical Analysis of Minerals. By Thomas Allan. 8vo.

Physiological Researches on Life and Death. By Xavier Bichat; translated from the French by F. Gold, Member of the Royal College of Surgeons, London. 8vo.—Longman and Co.

M. Portal has published a new edition of his Considerations sur les Maladies Hereditaires, avec Plusieurs Observations de l'Auteur, de celles de Professeur M. Mazzoni, & de M. Joseph Adams, M.D. de Londres.

---

### *Works preparing for the Press, &c.*

Dr. Pinckard is preparing for early publication, a new edition of the Notes on the West Indies, in two volumes, with considerable alterations and additions. The new matter will contain Remarks on the Islands of Martinique, Jamaica, and St. Domingo, also on the Condition and Treatment of the Slaves, and a Suggestion for effecting their Emancipation; together with additional Observations concerning the Seasoning, or Yellow Fever of the Tropical Regions.

Dr. Ronalds, of Coventry, is preparing for the press, a Translation of the celebrated little work of Cabanis, On Certainty in Medicine.

Sir James Fellowes intends publishing shortly, some Reports on the Pestilential Fever of Spain, being the Result of his Observations and Enquiries into the Origin and Progress of that Disorder in Andalusia in 1800, during a Residence of Five Years in that Country; and a particular detailed Account will be given of the Fatal Epidemic at Gibraltar in 1804, and of the two last at Cadiz in 1810 and 1813.

Preparing for re-publication, The Anatomical Plates of the Human Gravid Uterus, Size of Nature; by the late William and John Hunter; accompanied by accurate Descriptions. The Work has been very scarce.

In a few weeks will be ready for publication, General Anatomy applied to Physiology and Medicine; translated from Anatomie Generale, 4 tome, par Bichat, Physician to the Hospital of Humanity, and Professor of Anatomy and Physiology at Paris.

Mr. Grainger, surgeon of Birmingham, has a work in the press, on a New Mode of Opening the Bladder in certain Obstructions of the Urethra and Prostrate Gland; and on a simple Method of removing the Tonsils from the Throat, and other Tumours from the accessible Cavities of the Body.

Dr. Kentish, of Bristol, is at present preparing for the press, a new and greatly improved edition of his Essays on Burns.

---

### *NOTICES TO CORRESPONDENTS.*

*We are much obliged to Dr. Parry for his polite hint relative to one of our French articles. Our only apology must be, that we selected it from the American Journals before the original work came to hand. Several acceptable communications have been received.*

